# Efficacy and safety of ambrisentan in IgA nephropathy: real-world evidence

**DOI:** 10.3389/fimmu.2026.1967776

**Published:** 2026-10-29

**Authors:** Kuipeng Yu, Ke Zhao, Xingyu Chen, Fangli Wang, Sha Wang, Chunjie Wang, Fang Bai, Haiyun Li, Ling Guo, Xiangdong Yang

**Affiliations:** 1 Department of Nephrology, Qilu Hospital of Shandong University, Jinan, Shandong, China; 2 Department of Blood Purification, Qilu Hospital of Shandong University, Jinan, Shandong, China; 3 Department of Nephrology, Pingyuan County First People's Hospital, Dezhou, Shandong, China

**Keywords:** ACEI/ARB, ambrisentan, effectiveness, IgAN, safety

## Abstract

**Background:**

Ambrisentan, an endothelin receptor antagonist, reduces proteinuria; however, insufficient evidence is available to support its use in IgAN. The aim of this research is to investigate the efficacy and safety of ambrisentan in the treatment of IgAN nephropathy.

**Methods:**

This retrospective cohort study was enrolled 102 IgAN patients, which was confirmed by renal biopsy from Qilu Hospital, Shandong University (January 2021 to March 2026). 68 patients received ACEI/ARB treatment, while 34 IgAN patients received treatment with ACEI/ARB plus ambrisentan (5mg/day). The primary endpoint of the study assessed the percentage changes in UPCR in IgAN patients receiving different treatment regimens. Secondary endpoints included the change in eGFR and the safety of ambrisentan.

**Results:**

102 IgAN patients were enrolled in the research. ACEI/ARB + ambrisentan group exhibited higher rates of UPCR reduction compared with ACEI/ARB group: -35.9% [IQR, -46.7%, -12.2%] vs -11.8.0% [IQR, -35%, 3.19%] (P = 0.004) at 1 month, -52% [IQR, -61.6%, -26.6%] vs -22.4% [IQR, -42.5%, -2.8%] (P = 0.001) at 3 months, -43.1% [IQR, -61.3%, -27%] vs -29.8% [IQR, -52%, -0.2%] (P = 0.015) at 6 months. Furthermore, the UPCR was lower in the ACEI/ARB + ambrisentan group (0.45 g/g [IQR, 0.25, 0.85] vs 0.86 g/g [IQR, 0.52, 1.15]; P = 0.006) at 3 months, and (0.50 g/g [IQR, 0.37, 0.69] vs 0.85g/g [IQR, 0.51, 1.14]; P = 0.003) at 6 months. However, no statistically significant difference was observed between the two groups in terms of serum albumin levels. After treatment, the percentage change in eGFR from baseline was higher in the combination therapy group than in the ACEI/ARB group (1.6% vs -1.6%, p=0.014). At 6 months, the response rate in the ACEI/ARB + ambrisentan group was significantly higher (70.59% vs 48.53%, P = 0.034). Both groups experienced no serious adverse reactions.

**Conclusions:**

The addition of ambrisentan to renin-angiotensin-aldosterone system (RAS) blockers significantly reduces urinary protein in IgAN patients, while remaining relatively safe.

## Introduction

IgA nephropathy (IgAN) is the most common primary glomerular disease worldwide, and its long-term prognosis is relatively poor (1–3). Renin-angiotensin system inhibitors remain the first-line treatment for IgAN (2025 KDIGO Guidelines) (4). However, despite supporting treatments such as optimized renin-angiotensin system blockade, blood pressure control and lifestyle interventions, many patients still develop progressive proteinuria and declining renal function (5).

Proteinuria is an independent risk factor for progressive renal function decline and disease progression (6, 7). Glucocorticoids may be considered for patients with high-risk IgAN, but the risks and benefits must be weighed up (8–10). Therefore, new therapeutic strategies aimed at reducing proteinuria are urgently required for patients diagnosed with IgA nephropathy (11). Our previous research also investigated the therapeutic efficacy of MRA (Finerenone) in IgAN (12). Furthermore, several novel therapeutic agents, such as anticomplement [Iptacopan (13)], ERA(Atrasentan) (14), Nefecon (15), APRIL/BAFF inhibitors [Telitacicept (16)], and SGLT2i(Henagliflozin) (17) and so on, have demonstrated promising potential in the treatment of IgAN.

Ambrisentan, an endothelin receptor antagonist (ERA), has been approved for the treatment of pulmonary arterial hypertension (PAH) (18), and its safety has been demonstrated in numerous clinical trials and real-world studies. Theoretically, ERA can inhibit excessive ET-1 expression, alleviating renal damage (19). The latest 2.5-year follow-up data from the ALIGN Phase III trial demonstrate that atrasentan, the selective endothelin A receptor antagonist, reduces proteinuria and slows the rate of renal function decline in adult patients with IgA nephropathy (20). Recent research has discovered that ambrisentan possesses novel potential for the treatment of kidney disease (21–23).

However, high-quality clinical evidence on the efficacy of ambrisentan combined with ACEI/ARB inhibitors (ACEI/ARB+ ambrisentan) in the treatment of IgA nephropathy is still lacking. Therefore, the aim of this study is to investigate the efficacy and safety of ambrisentan in combination with ACEI/ARB inhibitors for the treatment of IgAN.

## Methods

### Study design and patients

This study included patients who were undergoing regular follow-up at the Department of Nephrology, Qilu Hospital of Shandong University between January 2021 and March 2026 and who had been diagnosed with IgA nephropathy via renal biopsy. Inclusion criteria: (1) Aged 18–65 years; (2) Primary IgA nephropathy confirmed by renal biopsy; (3) Baseline UPCR greater than 0.3 g/g; (4) Baseline eGFR greater than 30 ml/min/1.73 m²; (5) Complete clinical data throughout the follow-up period; Exclusion criteria:(1) Age under 18 years; (2) Secondary IgA nephropathy; (3) Baseline UPCR below 0.3 g/g; (4) Baseline eGFR below 30 ml/min/1.73 m²; (5) Patients with missing follow-up data; (6) Patients with IgAN who have undergone kidney transplantation. All IgAN patients are routinely treated with the maximum tolerated dose of ACEIs/ARBs. The maximum dose of ACEIs/ARBs is defined as: the highest dose recommended in Chinese prescribing information, or the maximum dose that the patient can tolerate without developing symptomatic hypotension, hyperkalaemia or an increase in serum creatinine levels of ≥30 per cent. This study has been approved by the Ethics Committee of Qilu Hospital, Shandong University (No. KYLL-2025-11-052).

### Date sources

The study utilized the electronic medical records database of Qilu Hospital, Shandong University, to extract inpatient and outpatient data on patients with IgAN nephropathy, primarily including demographic characteristics, diagnoses, histopathology reports (Renal Biopsy Report), laboratory test results and prescription records. The clinical data extracted primarily include: blood pressure, urinary protein-to-creatinine ratio (UPCR), serum creatinine, eGFR (calculated using the 2021 CKD-EPI creatinine equation), ALB, uric acid, hemoglobin, LDL-C, K and Na. Adverse events (AEs) occurring during treatment were also systematically recorded.

### Treatments

The ACEI/ARB group patients were treated with the maximum dose of ACEI/ARB; The ACEI/ARB + ambrisentan group received ambrisentan in addition to the maximum dose of ACEI/ARB. For IgAN patients who have received treatment with the maximum tolerated dose of an ACE inhibitor (ACEI) or angiotensin receptor blocker (ARB) for at least three months but continue to present with persistent proteinuria (urine protein-to-creatinine ratio [UPCR] ≥ 0.3 g/g) and an estimated glomerular filtration rate (eGFR) ≥ 30 mL/min/1.73 m², the addition of adjunctive therapy to standard treatment may be considered. Provided that the patient has no contraindications to endothelin receptor antagonists and has signed an informed consent form, prescribe ambrisentan as an adjunctive therapy. IgAN patients taking ambrisentan are required to undergo regular monthly follow-up examinations at hospital. Adjust the dosage and timing of ambrisentan and ACEI/ARB treatment appropriately according to the patient’s blood pressure.

### Efficacy assessment and outcomes

Significant response: UPCR reduced by ≥50% from baseline, and renal function remains stable (serum creatinine elevated by <20% from baseline). Effective response: UPCR reduced by 30% to 50% from baseline, and renal function was stable (serum creatinine increased by <20% from baseline). No response: Failure to achieve any of the above efficacy criteria. The primary endpoint is the percentage decrease in the urine protein-to-creatinine ratio (UPCR) from baseline to 6 months. Secondary endpoints included changes in estimated glomerular filtration rate (eGFR) and the safety of ambrisentan.

### Statistical analysis

The Shapiro-Wilk test was applied to assess the normality of the measurement data. In baseline data, continuous variables are presented as mean ± standard deviation or median (interquartile range), while categorical variables are presented as counts (percentages). Between-group differences were assessed using appropriate statistical tests. For continuous variables, either the Student’ s t-test or the Mann-Whitney U test is used, depending on the specific circumstances. For categorical variables, the chi-squared test or Fisher’s exact test is applied. The statistical significance threshold was set at P < 0.05. SPSS 25 and R 4.4.3 software are applied for data analysis.

## Results

### Patients disposition and baseline characteristics

Between January 2021 and March 2026, 102 IgAN patients were enrolled; among these, 68 patients received ACEI/ARB treatment, while 34 patients received ACEI/ARB in combination with ambrisentan (5 mg/day). The baseline characteristics of research participants are summarized in **Table 1**, and the distribution of the Oxford Pathology Grading was generally fairly balanced between the two groups (**Table 2**). The levels of proteinuria among the IgAN patients recruited for the study were predominantly mild to moderate; the UPCR in ACEI/AR group was 0.98 (0.75, 1.67) g/g, and the UPCR in ACEI/ARB + ambrisentan group was 0.86 (0.64, 1.46) g/g. There were no significant differences in the baseline data between the two groups (P > 0.05). The flowchart for this study appears in **Figure 1**.

**Table 1:** Baseline characteristics of the IgA nephropathy patients.

Characteristics	ACEI/ARB group(n=68)	ACEI/ARB + Ambrisentan group(n=34)	P Value
Age (years)	39.53 ± 9.99	38.97 ± 10.32	0.793
Male sex (n, %)	35(51.5%)	19(55.9%)	0.674
BMI(kg/m^2^)	24.75(22.42, 28.40)	23.50(22.10, 25.50)	0.172
SBP (mmHg)	132.71 ± 20.43	138.15 ± 26.62	0.256
DBP (mmHg)	83.57 ± 13.58	88.32 ± 18.56	0.145
Glucocorticoids or Immunosuppressive therapy treatment (n, %)	27(39.7%)	13(38.2%)	0.653
CTX	3	1	0.718
MMF	4	3	0.58
TAC	3	2	0.746
HCQ	4	3	0.58
Leflunomide	2	0	0.313
Telitacicept	5	2	0.782
SGLT-2i	20(29.4%)	9(26.5%)	0.756
Nefecon	9(13.2%)	4(12.7%)	0.834
UPCR(g/g)	0.97(0.75, 1.67)	0.86(0.64, 1.61)	0.484
Scr(μmol/L)	87.50(66.5, 113.71)	86.50(75.75, 115.50)	0.647
eGFR(ml/min/1.73m^2^)	85.74(61.00, 105.12)	92.50(62.18, 102.33)	0.837
eGFR≥45ml/min/1.73m^2^	62(91.2%)	34(94.1%)	0.602
eGFR<45ml/min/1.73m^2^	6(8.8%)	2(5.9%)	
Hb(g/L)	129.62 ± 19.80	136.44 ± 15.47	0.082
ALB level(g/L)	42.58 ± 3.65	42.21 ± 4.39	0.66
UA(mmol/L)	338.25 ± 69.68	329.50 ± 63.29	0.539
LDL-C(mmol/L)	2.95 ± 1.04	3.26 ± 1.11	0.154
K(mmol/L)	4.08 ± 0.34	4.10 ± 0.33	0.743
Na(mmol/L)	141.37 ± 2.43	141.97 ± 1.77	0.202

Data are shown as n (%), n (%; 95% CI), or median (IQR). BMI, body mass index; SBP, Systolic blood pressure; DBP, Diastolic Blood Pressure; eGFR, estimated glomerular filtration rate; UPCR, urine protein-creatinine ratio.

**Table 2:** Oxford classification of IgA nephropathy.

Oxford classification	ACEI/ARB group(n=68)	ACEI/ARB + Ambrisentan group(n=34)	P value
M0	31(45.6%)	11(32.4%)	0.2
M1	37(54.4%)	23(67.6%)	
E0	24(35.3%)	15(44.1%)	0.387
E1	44(64.7%)	19(55.9%)	
S0	6(8.8%)	6(17.6%)	0.192
S1	62(91.2%)	28(82.4)	
T0	22(32.4%)	17(50%)	0.104
T1	30(44.1%)	14(41.2%)	
T2	16(23.5%)	3(8.8%)	
C0	41(60.3%)	16(47.1%)	0.426
C1	26(38.2%)	17(50%)	
C2	1(1.5%)	1(2.9%)	

**Figure 1:**
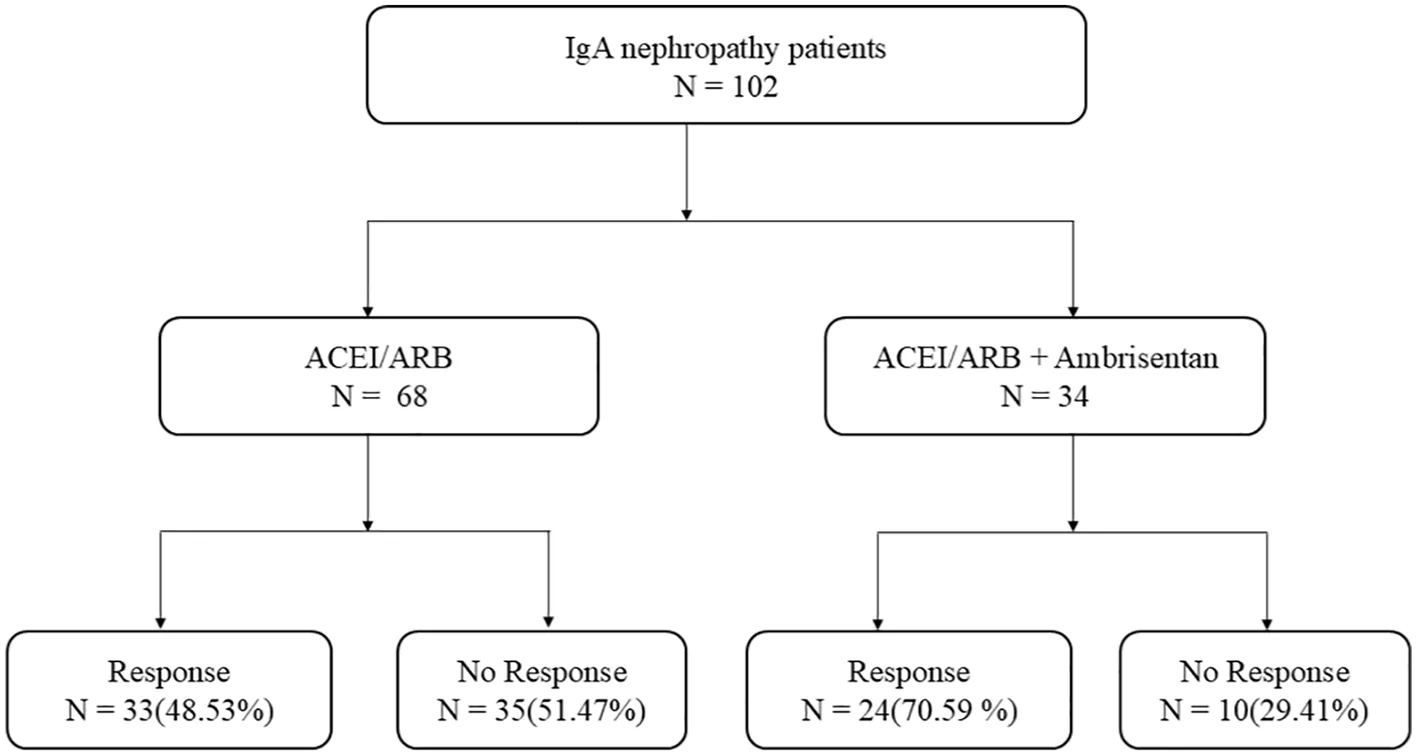
Flow chart of the study.

### Research outcomes

**Table 3** demonstrates the efficacy outcome measure indicators for the ACEI/ARB+ ambrisentan group compared with the ACEI/ARB group. The ACEI/ARB + ambrisentan group achieved significantly greater percentage reductions than ACEI/ARB group:-35.9% [IQR, -46.7%, -12.2%] vs -11.8.0% [IQR, -35%, 3.19%] (P = 0.004) at 1 month, -52% [IQR, -61.6%, -26.6%] vs -22.4% [IQR, -42.5%, -2.8%] (P = 0.001) at 3 months, -43.1% [IQR, -61.3%, -27%] vs -29.8% [IQR, -52%, -0.2%] (P = 0.015) at 6 months. During the 6-month follow-up, the UPCR was lower in the ACEI/ARB + ambrisentan group (0.45 g/g [IQR, 0.25, 0.85] vs 0.86 g/g [IQR, 0.52, 1.15]; P = 0.006) at 3 months, and (0.50 g/g [IQR, 0.37, 0.69] vs 0.85g/g [IQR, 0.51, 1.14]; P = 0.003) at 6 months (**Figure 2B**). Taking into account the impact of the higher baseline eGFR and fewer T2 lesions in the ambrisentan group, we also conducted a supplementary analysis using an analysis of covariance (ANCOVA) model; the results of the full analysis are presented in **Supplementary Table 1**. In addition, we also comparatively analyzed changes in serum albumin levels. No significant difference was observed in serum albumin levels between the two groups after 6 months of treatment (**Supplementary Figure 4**). Similarly, after 6 months, eGFR levels (**Supplementary Figure 5**) and serum creatinine (**Supplementary Figure 6**) and were approximately equivalent between the two groups, with no statistically significant differences observed in either parameter (P>0.05). However, the percentage change in eGFR from baseline was higher in the combination therapy group than in the ACEI/ARB group (1.6% vs -1.6%, p=0.014, **Figure 2C**). Furthermore, **Figure 3** shows that the response rate in the ACEI/ARB + ambrisentan group was significantly higher (70.59% vs 48.53%, P = 0.034, **Table 4**).

**Table 3:** Comparison of outcomes between the ACEI/ARB + Ambrisentan and ACEI/ARB groups.

Variables	Time	ACEI/ARB group(n=68)	ACEI/ARB + Ambrisentan group(n=34)	P value
UPCR(g/g)	Baseline	0.98(0.75,1.67)	0.86(0.64,1.46)	0.433
	1 month	0.84(0.47,1.75)	0.55(0.32,1.40)	0.060
	3 month	0.86(0.52.1.15)	0.45(0.25,0.85)	0.006**
	6 month	0.85(0.51,1.14)	0.50(0.37,0.69)	0.003**
Percentage change of UPCR(%)	1 month	-11.8(-35.3,19)	-35.9(-46.7, -12.2)	0.004**
	3 month	-22.4(-42.5,-2.8)	-52.0(-61.6, -26.6)	0.001**
	6 month	-29.8(-52,0)	-43.1(-61.3,-27.0)	0.015*
Scr(μmol/L)	Baseline	87.5(66.5, 113.75)	86.5(75.75, 115.5)	0.647
	1 month	90.5(76.0,114.25)	83(72.75,106.75)	0.322
	3 month	94.0(78.25,113.0)	86.5(73.75,105.5)	0.347
	6 month	93.5(77,113.65)	84.5(74.75,99.5)	0.131
eGFR(ml/min/1.73m2)	Baseline	85.8(61.0,105.1)	92.51(62.2,102.3)	0.837
	1 month	83.2(58.2,99.0)	90.4(58.2,104.7)	0.493
	3 month	85.8(58.1,99.1)	87.5(62.5,101.7)	0.915
	6 month	83.9(55.9,99.8)	93.7(72.9,103.5)	0.225
Percentage change of eGFR (%)	1 month	-2.2(-5.8,0.97)	0.21(-6.7,4.3)	0.134
	3 month	-1.4(-7.7,2.2)	-5.0(-10,8,2.9)	0.258
	6 month	-1.6(-8.5,2.3)	1.6(-3.9,10.9)	0.014*
ALB	Baseline	42.48 ± 3.65	42.21 ± 4.56	0.660
	1 month	42.63 ± 3.68	42.5 ± 4.56	0.873
	3 month	42.85 ± 3.39	42.57 ± 4.36	0.725
	6 month	42.94 ± 3.22	43.03 ± 3.70	0.892
Percentage change of ALB (%)	1 month	0.28 ± 6.08	0.88 ± 7.36	0.663
	3 month	0.89 ± 6.55	1.07 ± 6.71	0.897
	6 month	1.11 ± 6.28	2.82 ± 13.12	0.375
Hg(g/L)	Baseline	129.61 ± 2.4	136.44 ± 2.65	0.082
	1 month	132.72 ± 2.01	134 ± 3.42	0.732
	3 month	131.52 ± 2.32	135.82 ± 3.34	0.296
	6 month	133.73 ± 1.90	137.5 ± 3.01	0.275
UA(μmol/L)	Baseline	338.25 ± 8.45	326.55 ± 10.51	0.408
	1 month	357.30 ± 10.55	351.11 ± 8.83	0.703
	3 month	339 ± 9.34	320.94 ± 11.40	0.246
	6 month	358.67 ± 7.54	348.47 ± 9.55	0.421
LDL-C(mmol/L)	Baseline	2.94 ± 0.13	3.20 ± 0.18	0.236
	1 month	2.94 ± 0.10	3.02 ± 0.17	0.648
	3 month	3.20 ± 0.13	2.91 ± 0.13	0.158
	6 month	3.00 ± 0.10	2.91 ± 0.11	0.585
Na(mmol/L)	Baseline	141.36 ± 0.29	141.97 ± 0.30	0.202
	1 month	140.98 ± 0.35	141.38 ± 0.55	0.529
	3 month	140.89 ± 0.31	140.44 ± 0.56	0.445
	6 month	141.30 ± 0.34	141.58 ± 0.34	0.600
sK(mmol/L)	Baseline	4.08 ± 0.04	4.10 ± 0.06	0.743
	1 month	4.22 ± 0.04	4.23 ± 0.04	0.980
	3 month	4.18 ± 0.05	4.21 ± 0.07	0.721
	6 month	4.26 ± 0.04	4.28 ± 0.06	0.869

**Figure 2:**
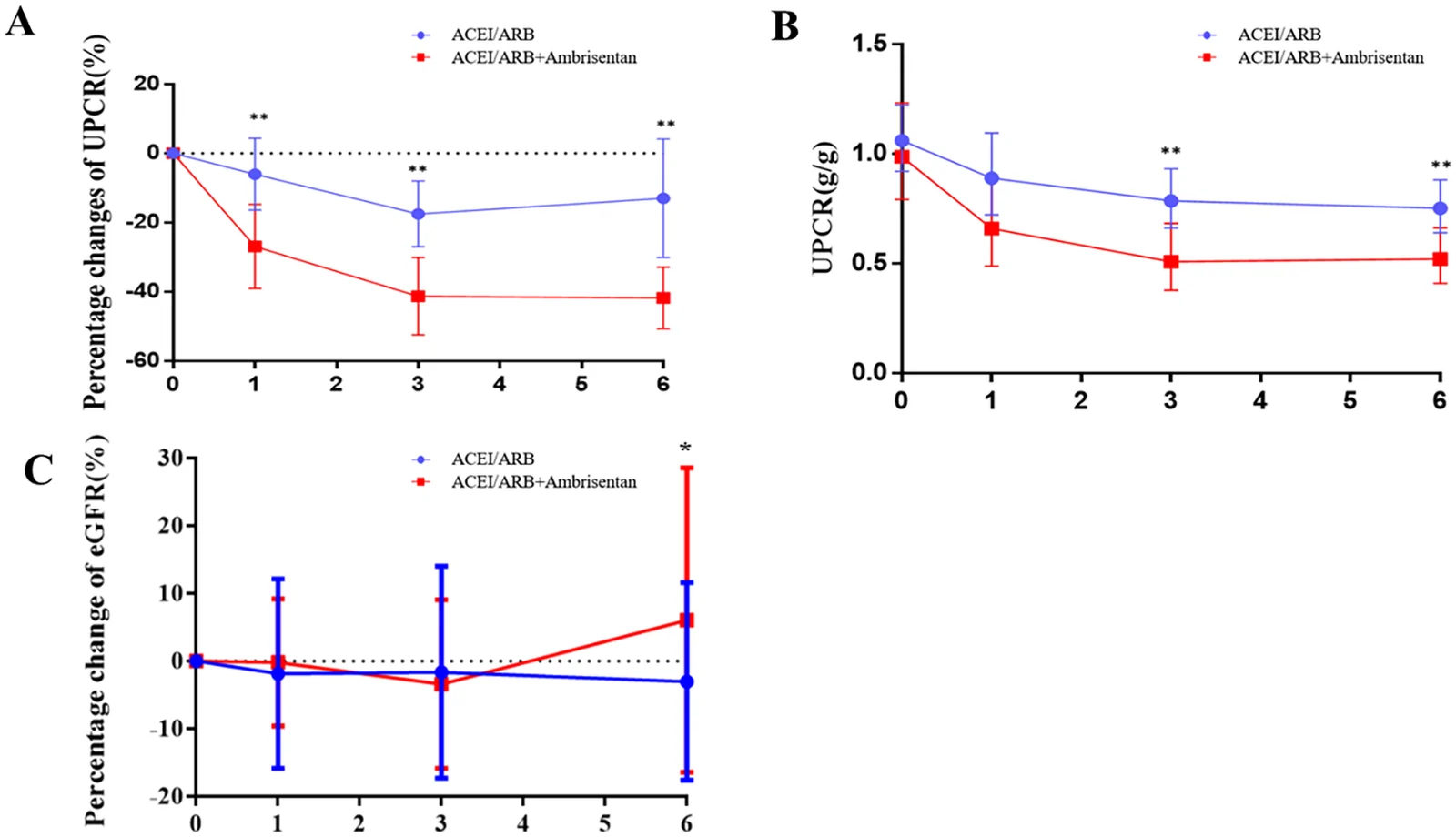
Efficacy outcome changes during treatment with ACEI/ARB versus ACEI/ARB plus ambrisentan. **(A)** The percent change in UPCR from baseline; **(B)** The change of UPCR; **(C)** The percent change in eGFR from baseline. *P<0.05, **P<0.01.

**Figure 3:**
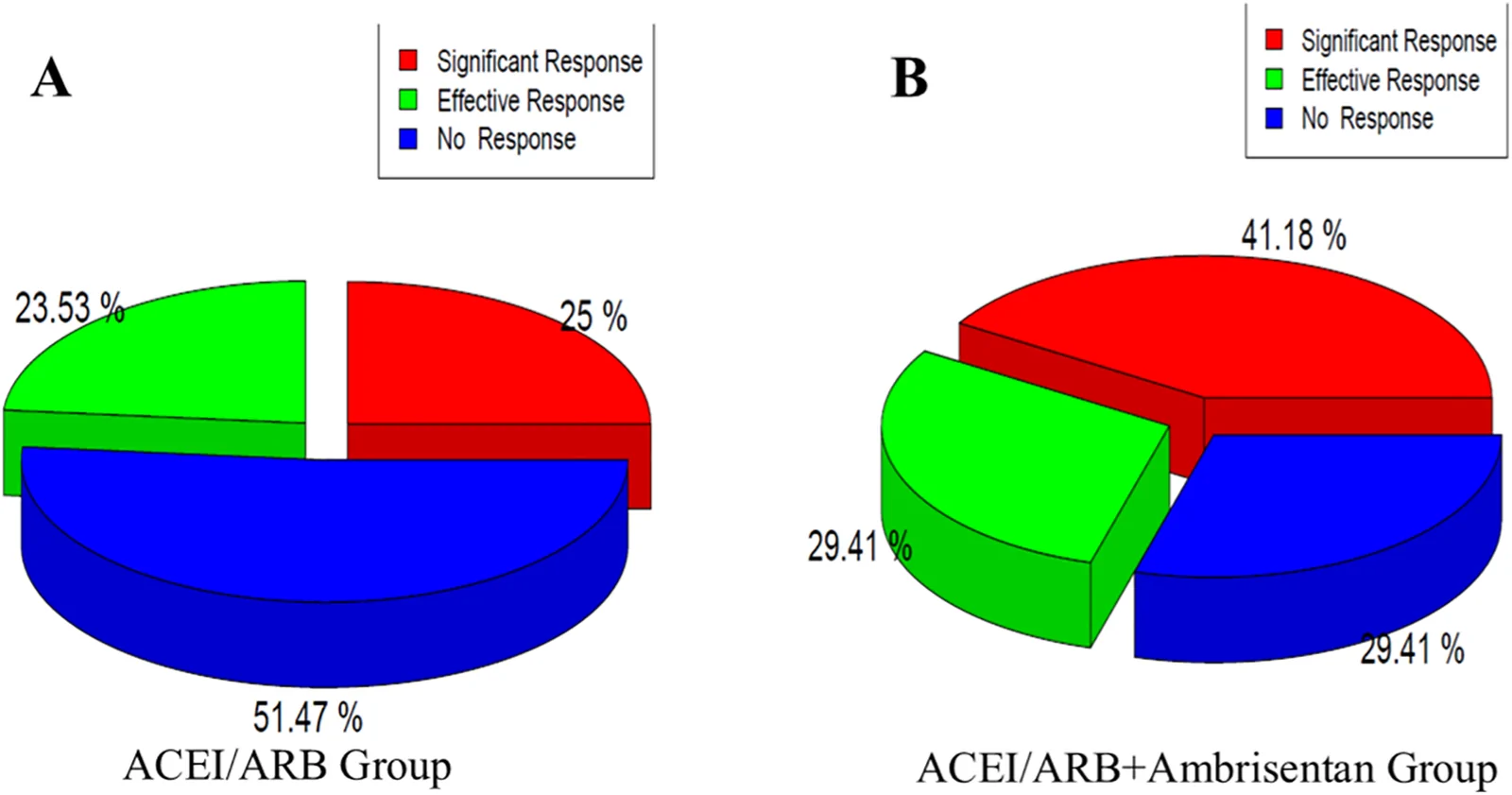
Comparison of the treatment response rates between the two groups. **(A)** The treatment response rates in the ACEI/ARB group. **(B)** The treatment response rates in the ACEI/ARB + ambrisentan group.

**Table 4:** Comparison of the treatment response rate between the ACEI/ARB + Ambrisentan and ACEI/ARB groups.

Characteristic	Number	Significant response	Effective response	No response	All response
ACEI/ARB group	68	17(25%)	16(23.53%)	35(51.47%)	33(48.53%)
ACEI/ARB+Ambrisentan group	34	14(41.18%)	10(29.41%)	10(29.41%)	24(70.59%)
P value					0.034

### Safety outcomes

Adverse events are summarized in **Table 5**. Notably, the most common adverse reactions in the ACEI/ARB + ambrisentan group were hypotension and peripheral oedema. Patients with hypotension experienced improvements in their blood pressure following an adjustment to the ACEI/ARB dose or by staggering the timing of administration with ambrisentan. The five patients who exhibited peripheral oedema had mild symptoms, and none experienced any serious episodes of peripheral oedema.

**Table 5:** Adverse events.

SAEs	ACEI/ARB group(n=68)	ACEI/ARB + Ambrisentan group(n=34)	P value
SAE(n, %)	0(0)	0(0)	–
others			
Hypotension	4	5	0.459
Peripheral edema(n, %)	3	5	0.116
Headache(n, %)	0	2	NA
Heart palpitations	0	0	NA
Elevated hepatic enzyme(n, %)	0	0	NA

Additionally, two patients taking ambrisentan developed headache. In summary, no serious adverse events were observed in either group of patients during the treatment period.

## Discussion

Recent research reveals that the endothelin system is one of the critical systems involved in the progression of IgA nephropathy (5, 24). Endothelin-1 (ET-1) expression in the kidneys of patients with IgAN. Highly expressed ET-1, by activating the endothelin type A receptor (ETA), mediates intrarenal vasoconstriction, mesangial cell proliferation and podocyte damage, consequently leading to increased proteinuria, renal inflammation and fibrosis, and accelerating the deterioration of renal function (25).

Endothelin receptors, particularly the activation of ERA, are recognized critical factors in the progression of chronic kidney disease. Ambrisentan, high selectivity in antagonizing the ETA receptor, demonstrates the capacity to block the binding of endothelin-1 (ET-1) to the ETA receptor (26). This, in turn, has been shown to improve renal microvascular circulation, consequently reducing proteinuria and slowing the progression of renal dysfunction (27, 28). Ambrisentan has been applied in the treatment of pulmonary arterial hypertension (29) and currently demonstrates new potential in the treatment of kidney disease.

During the 6-month observational period in this single-center retrospective study, treatment with ambrisentan in combination with ACEI/ARB demonstrated more significant reductions in proteinuria levels compared with the ACEI/ARB group. At 6 months, all the response rate the ambrisentan + ACEI/ARB group was 70.58% (**Figure 3**). More importantly, the research observed that the percentage decrease in UPCR in the ambrisentan + ACEI/ARB group was significantly lower than that in the ACEI/ARB group at one month [−35.9% (−46.7, −12.2) vs. −11.8% (−35.3, 3.19), p =0.004]. After 6 months of treatment, the ambrisentan + ACEI/ARB group of the percentage change in UPCR had decreased by 43.1% (-61.3%, -26.6%) from baseline (**Table 3**, **Figure 2A**). However, the median UPCR reduction in the combination group was greater at 3 months (52.0%) than at 6 months (43.1%), whereas the control group showed continued improvement from 3 to 6 months (22.4% to 29.8%). Several explanations are possible. First, the antiproteinuric effect of ambrisentan may peak early and partially attenuate thereafter. Second, regression to the mean and natural fluctuation in UPCR may contribute. Third, changes in concomitant therapies, ACEI/ARB dose adjustment due to hypotension, fluid retention, dietary sodium intake, and adherence may differ between groups. Finally, the small sample size limits precise estimation of time trends.

Consistent with the results of our study, Professor Zhang Hong’s research found that the combination of ambrisentan and empagliflozin for the treatment of IgAN can significantly reduce urinary protein levels in patients with IgAN, preserve renal function and reduce fluid retention (23). Furthermore, ambrisentan has exhibited favorable efficacy in decreasing urinary protein levels and acceptable safety in Alport Syndrome patients (22). New research has discovered that atrasentan, which is also a selective ETA antagonist, has now been evaluated in the phase 3 ALIGN trial. The results of the research indicate that treatment with atrasentan in IgAN patients reduced proteinuria and preserved renal function after 2.5 years (20). At the same time, the PROTECT trial also demonstrated that sparsentan, a dual endothelin-angiotensin receptor antagonist, significantly reduced proteinuria and improved the chronic decline in eGFR in IgAN patients (16, 30). These trials support the endothelin pathway as a therapeutic target in IgAN. However, the clinical evidence regarding the use of ambrisentan for the treatment of IgAN remains relatively limited, and further prospective clinical trials are still required to validate its efficacy and safety.

New research guidelines recommend that proteinuria in patients with IgAN be controlled to ≤0.3 g/day or the urine protein-to-creatinine ratio to ≤0.2 g/g, while simultaneously ensuring that eGFR remains stable (with an annual decline of ≤1 ml/min) and that persistent trace hematuria is resolved (4, 31). In our study, 13 patients (38.2%) in the ambrisentan combined with ACEI/ARB group were treated with glucocorticoids or other conventional immunosuppressants. In addition, four patients in the ACEI/ARB + ambrisentan group had previously received or were concurrently receiving Nefecon (targeted-release budesonide), and the treatment regimens provide some degree of improvement in the condition of patients with IgAN. In the phase 3 NefIgArd trial, 9 months of Nefecon 16 mg/day significantly reduced proteinuria and slowed kidney function decline compared with placebo in patients with IgAN at high risk of progression despite optimized RASi. In our cohort, Nefecon-treated patients had persistent proteinuria above the desired threshold at baseline despite Nefecon exposure. The addition of ambrisentan led to a further reduction in UPCR in these patients, suggesting that endothelin receptor antagonism may provide additional antiproteinuric benefit beyond that achieved with Nefecon. However, the small number of Nefecon-treated patients in each group and the retrospective design preclude any definitive conclusion.

Notably, 41.18% (14/34) of patients achieved significant response after 6 months. We attempted to conduct a subgroup analysis stratified by baseline eGFR (by baseline eGFR (≥45 vs <45 mL/min/1.73 m²) to assess the anti-proteinuric effect of amblisentan. However, due to the small number of patients with an eGFR < 45 (n = 2 in the ACEI/ARB plus amblisentan group), it was not possible to perform a meaningful statistical analysis (**Supplementary Table 2**). Furthermore, the subgroup analysis revealed that, when stratified according to the Oxford T-score, the UPCR in patients with T0-stage IgAN decreased significantly within one month. Given the small sample size for the T-stratification in this study, this observation requires validation in a larger cohort (**Supplementary Table 3**). More importantly, however, regardless of whether an SGLT2 inhibitor was used as baseline therapy, the anti-proteinuric effect of ambrisentan appeared to remain consistent, with no significant statistical difference observed; this may be related to the small number of patients receiving SGLT2 inhibitors (**Supplementary Figure 7**).Our research also discovered that, at the 6-month mark, although no significant change in eGFR was observed in the ACEI/ARB + ambrisentan group, the percentage change in eGFR showed an upward trend (**Figure 2C**). At month 6, the percentage change in eGFR from baseline was higher in the combination therapy group than in the ACEI/ARB group, and this difference was statistically significant (1.6% vs –1.6%, P = 0.014). However, the difference in absolute eGFR levels between the two groups at month 6 was not statistically significant. Given the short duration of follow-up, further studies with longer follow-up periods are required, using eGFR slope and renal hard endpoints as primary outcome measures, to further evaluate the long-term renal protective effects of ambrisentan in patients with IgAN.

Throughout the entire study period, IgAN patients generally tolerated ambrisentan well, and no serious adverse events were reported (**Table 5**). In our study, the main adverse reaction was sodium and water retention and hypotension, which was mostly mild. The IgAN patients’ symptoms improved following adjustments to the drug dosage and the timing of administration of both drugs. In addition, two patients experienced headache. Anemia has been recognized as the most common adverse reaction to ambrisentan (21, 23, 32). Professor Zhao, Jinghong’s research also observed that, following three months of treatment with ambrisentan, 24.3% of patients developed anemia, the majority of which was mild or moderate (21). However, our research suggests that the occurrence of anemia may be closely related to patients’ baseline renal function; however, further large-scale studies are required to determine whether ambrisentan exacerbates anemia in IgAN patients.

In summary, our study preliminarily explored the efficacy and safety of ambrisentan in combination with an ARB in patients with IgAN; however, there remain many limitations. To begin with, this research is single-center and retrospective, with a relatively small sample size. In addition, the absence of certain baseline data (such as the time of diagnosis and data on trace hematuria) may introduce residual confounding factors. Furthermore, the follow-up period for IgAN patients was relatively short (6 months), making it impossible to assess the long-term renal benefits and safety outcomes of ambrisentan treatment for IgAN. In some IgAN patients, the concomitant use of drugs such as glucocorticoids or other immunosuppressants, SGLT2 inhibitors or Nefecon may influence the study results. Consequently, more large-scale, multicenter, prospective clinical trials are urgently required to assess the long-term efficacy and safety of ambrisentan in the treatment of IgAN.

## Conclusion

To summarize, the combination of ambrisentan with an ACEI/ARB significantly decreases proteinuria, showing safety outcomes. Consequently, the combination may represent an emerging potential treatment option for IgAN.

## Data Availability

The original contributions presented in the study are included in the article/**Supplementary Material**. Further inquiries can be directed to the corresponding authors.
