# Supplementary material for: Efficacy and safety of ambrisentan in IgA nephropathy: real-world evidence
Source: Front Immunol. 2026 Oct 29;17:1967776. doi: 10.3389/fimmu.2026.1967776 (PMC13754828; doi:10.3389/fimmu.2026.1967776)
Supplement: Supplementary file 1 [file SupplementaryFile1.zip › Supplementary material/Supplementary material.doc]

**Supplementary Table**

**Table 1 Results of the analysis of covariance (ANCOVA) for UPCR at months 1, 3 and 6**

| **Time** | **Adjusted geometric mean ratio** | **P-value** | **Adjusted absolute difference (g/g)** | **P-value** |
| --- | --- | --- | --- | --- |
| 1 Month | **0.76（95% CI 0.60–0.96）** | **0.022** | **−0.19（95% CI −0.36, −0.02）** | **0.022^*^** |
| 3 Month | **0.63（95% CI 0.49,0.80）** | **0.0003** | **−0.28（95% CI −0.44, −0.12）** | **0.0003^***^** |
| 6 Month | **0.71（95% CI 0.56,0.90）** | **0.005** | **−0.19（95% CI −0.34, −0.04）** | **0.005^**^** |

Adjusted UPCR: ACEI/ARB + Ambrisentan group vs ACEI/ARB group adjusted GMR (Adjusted geometric mean ratio)

**Table 2 Subgroup analysis by baseline eGFR group (≥45 vs <45 mL/min/1.73 m²)**

| **Subgroup** | **Time**  **(month)** | **ACEI/ARB（n=62）** | **ACEI/ARB + Ambrisentan（n=34）** | **Z** | **P-value** |
| --- | --- | --- | --- | --- | --- |
| eGFR ≥45  UPCR（g/g） | 0 | 0.89(0.72,1.42) | 0.86(0.66,1.41) | -0.271 | 0.786 |
|  | 1 | 0.79(0.46,1.31) | 0.55(0.31,1.40) | -1.281 | 0.200 |
|  | 3 | 0.76(0.46,1.22) | 0.43(0.24,0.91) | -2.354 | 0.019^**^ |
|  | 6 | 0.83(0.47,1.12) | 0.49(0.35,0.76) | -2.434 | 0.015^**^ |

**Table 3 Subgroup analysis by Oxford T score**

| **Subgroup** | **Time**  **(month)** | **ACEI/ARB（n= 22）** | **ACEI/ARB + Ambrisentan（n=17）** | **Z** | **P-value** |
| --- | --- | --- | --- | --- | --- |
| T0  UPCR（g/g） | 0 | 0.67(0.45,0.91) | 0.51(0.28,0.83) | -1.672 | 0.095 |
|  | 1 | 0.57(0.34,0.86) | 0.35(0.26,0.51) | -2.38 | 0.017^*^ |
|  | 3 | 0.65(0.34,1.00) | 0.30(0.21,0.71) | -2.068 | 0.039^*^ |
|  | 6 | 0.47(0.24,0.72) | 0.31(0.19,0.56) | -2.89 | 0.003^**^ |
| T1/T2  UPCR（g/g） | 0 | 1.24(0.75,1.95) | 0.81(0.54,2.92) | -0.372 | 0.71 |
|  | 1 | 1.07(0.57,2.31) | 0.65(0.30,2.34) | -1.014 | 0.31 |
|  | 3 | 1.03(0.54,1.72) | 0.46(0.24,2.93) | -1.611 | 0.107 |
|  | 6 | 1.02(0.53,1.56) | 0.44(0.18,0.96) | -2.416 | 0.016^*^ |
